# Genomic Medicine Among Ophthalmologists: Knowledge, Current Practice, and Barriers

**DOI:** 10.3390/jpm16050267

**Published:** 2026-05-16

**Authors:** Walaa Bakhamees, Hend Alsafran, Hani Basher ALBalawi, Naif M. Alali, Yousef A. Alotaibi, Moustafa S. Magliyah

**Affiliations:** 1 Vitreoretinal and Uveitis Division, King Khaled Eye Specialist Hospital, Riyadh 11462, Saudi Arabia; wbakhamees@uj.edu.sa (W.B.); hend_alsafran@hotmail.com (H.A.); mussam8@yahoo.com (M.S.M.); 2 Division of Ophthalmology, Department of Surgery, College of Medicine, University of Jeddah, Jeddah 21589, Saudi Arabia; 3 Mohamad Al Dakhan Eye Center, AL-Amiri Hospital, Ministry of Health, Kuwait City 13110, Kuwait; 4 Division of Ophthalmology, Department of Surgery, Faculty of Medicine, University of Tabuk, Tabuk 71491, Saudi Arabia; nmalali@ut.edu.sa; 5 Department of Ophthalmology, College of Medicine, King Khalid University, Abha 61421, Saudi Arabia; yalotaibi@kku.edu.sa

**Keywords:** knowledge, attitude, practices, genomic medicine, ophthalmologists

## Abstract

**Background/Objectives**: To assess the knowledge, attitudes, and practices of ophthalmologists in Saudi Arabia towards genomic medicine and genetic testing, in light of the growing significance of genomics in ophthalmology and the national transition towards precision medicine. **Methods**: A cross-sectional, questionnaire-based survey was conducted among ophthalmologists, including consultants, specialists, fellows, and residents, across Saudi Arabia. The questionnaire included four domains: demographics, knowledge of genomic principles and gene therapy, self-rated confidence in genetic tasks (scored 1–10), and attitudes toward genetic testing. Data were analyzed using descriptive and inferential statistics, with subgroup comparisons performed using chi-square tests and *t*-tests/ANOVA. **Results**: A total of 115 ophthalmologists participated (46% male, 54% female; mean age 34 years; mean post-board experience 4 years). Most were consultants (40%) and practiced in Riyadh (52%). Knowledge was variable: 92% correctly identified human chromosome count, and 99% recognized autosomal recessive inheritance, but only 9% answered DNA base-pairing correctly, and 54% recognized mitochondrial inheritance. Confidence was highest for referral to specialists (mean 7.3/10) and lowest for test selection and counseling (4.7/10). The internet was the primary knowledge source among our sample (65%). The majority of individuals had positive attitudes towards genomic medicine: 90% believed testing was beneficial, 89% considered it enhanced health outcomes, and 89% indicated they would undergo testing themselves. On the other hand, 77% indicated difficulty in access, 91% strongly concurred on the significance of privacy and confidentiality, and more than half expressed concerns regarding misuse and bias. **Conclusions**: Ophthalmologists in Saudi Arabia acknowledge the importance of genetics. Yet, there are substantial gaps in knowledge and familiarity with genomic medicine and genetic testing. To overcome these challenges, it is essential to integrate genetics into ophthalmology curricula.

## 1. Introduction

Genomic medicine is an evolving part of healthcare today and integrates genetic and molecular data into clinical decision-making to improve disease prevention, diagnosis, and treatment. In ophthalmology, genomic medicine improves diagnostic accuracy and facilitates the adoption of a personalized approach for numerous diseases that have a genetic basis, such as retinal dystrophies, congenital glaucoma, and hereditary corneal disorders. Despite these developments in the medical field, research from different countries continues to show significant gaps in the understanding of this field amongst healthcare professionals [1,2,3,4]. Such gaps in understanding genomic medicine might affect patient care through delayed referrals to ophthalmic genetic services, delayed genetic testing, and absent or deficient counseling for family members against consanguinity. Surveys of medical students and physicians in the Arab countries revealed predominantly favorable opinions towards genomic medicine, although they highlighted a lack of formal education and practical training. In these studies, healthcare professionals recognized insufficient training and interpretative abilities as significant obstacles in integrating genomics in their practice [5]. Research in Saudi Arabia has demonstrated similar observations; pharmacists and physicians have shown positive attitudes about genetic medicine, but exhibited confusion regarding the application of test results in clinical practice [6,7]. In ophthalmology, genetic testing and gene-based therapies are rapidly evolving, making it critical that physicians in these specialties have a good background and understanding of this rapidly evolving field. Ophthalmologists need to be aware that gene therapy is transforming the treatment of various ophthalmic conditions by addressing the root causes of debilitating diseases like inherited retinal dystrophies [8]. In addition, ophthalmology is one of the leading specialties in the clinical applications of gene therapy, and appropriate referral by ophthalmologists for genetic testing directly impacts eligibility for emerging treatments. In a country with high rates of consanguinity like Saudi Arabia, the accessibility to ophthalmic genetic services and the availability of genetic testing with meaningful interpretation might reflect positively on medical care for affected patients, as well as for other family members through early prevention and premarital genetic counseling. Ethical, legal, and societal issues—such as the proper use of genetic information, possible discrimination, and patient confidentiality—however, remain important considerations [9,10]. In the era of personalised medicine, identification of gaps in the knowledge and understanding of genomic medicine among physicians and ophthalmologists facilitates the transition to personalised medicine. The availability of more genomic information and molecular mechanisms of inherited diseases leads to a better understanding of disease and higher-quality care provided to patients. Improving the practices and applications of genomic medicine among ophthalmologists through proper identification of patients, utilization of genetic testing to yield comprehensive genomic information, and appropriate patient referral represent integral steps towards personalised patient care. Our study aims to evaluate the knowledge, attitudes, and practices of ophthalmologists in Saudi Arabia towards genomic medicine and genetic testing, given the evolving significance of genomics in ocular care. It also assesses demographic factors, understanding of genetics and gene therapy, self-reported trust in genetic counseling and testing, and perspectives on the clinical and ethical aspects of genetic medicine.

## 2. Materials and Methods

This study was a cross-sectional, questionnaire-based study among ophthalmologists in Saudi Arabia. Eligible participants were ophthalmology consultants, specialists, fellows, and residents. The survey was distributed electronically to ensure accessibility across all regions of the Kingdom. The study was approved by IRB at KKESH as the Saudi Ophthalmology Society (SOS) has no IRB. The SOS has ophthalmologists’ contacts, not the collected data, because data collection was performed based on the response from ophthalmologists in this study. The questionnaire (Table S1) was modified from recognized questionnaires used to evaluate knowledge, attitudes, and practices in genomic medicine and tailored for the ophthalmology domain. It consisted of four domains: (1) demographics, (2) understanding of genetic principles, inheritance mechanisms, and gene therapy, (3) self-rated confidence in performing genomics-related tasks such as family history assessment, test interpretation, and counseling, and (4) attitudes and perceptions regarding genomic testing, its availability, benefits, and ethical concerns. To test the understanding of genetic principles, inheritance mechanisms, and gene therapy, questions were modified from a survey that was provided for healthcare professionals [11]. To test self-rated confidence to perform genomics-related tasks, questions were modified from a survey that was provided for primary care physicians [12]. Both sets of questions were modified to more appropriately address ophthalmologists. The face validity of the survey was assessed by ten ophthalmologists to ensure the clarity and the premise of each question. The survey was then piloted for content, design, readability, and comprehension by ten different ophthalmologists. The required amendments were made based on the feedback to develop the final survey. The survey was distributed using professional ophthalmology email lists and contacts between June and August 2025. All ophthalmologists who had active memberships in the Saudi Ophthalmology Society (SOS) were invited to participate in the study. Ophthalmologists who did not have active SOS memberships were excluded. The total number of ophthalmologists who were invited was 429 ophthalmologists. Informed consent was obtained electronically prior to survey completion. The data extraction from the responses was performed electronically through predefined variables and was then transferred to the IBM Statistical Package for Social Sciences (SPSS). The SPSS version 30.0.0 was used to perform the statistical analysis. Descriptive statistics were generated for categorical variables (frequencies, percentages) and continuous variables (means, standard deviations, medians, ranges). Knowledge questions were analyzed for correctness; confidence scores are summarized as mean ± SD. Attitudinal responses are reported as proportions.

## 3. Results

**Figure 1:**
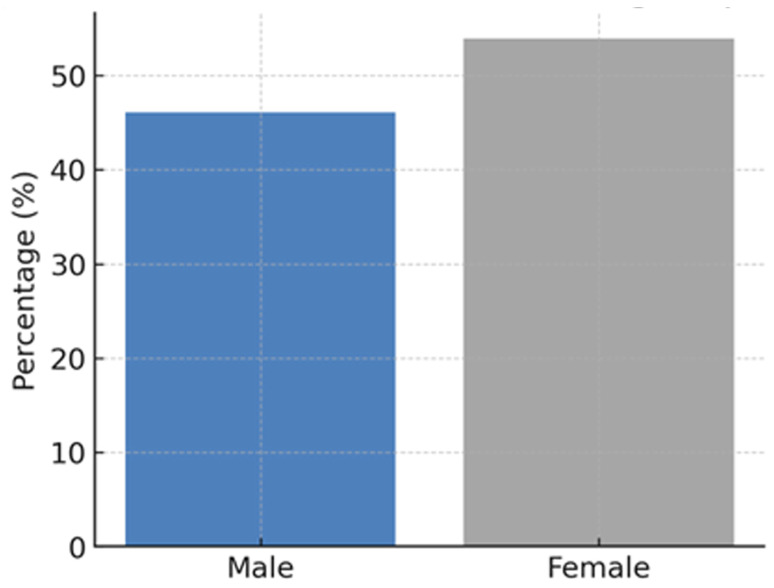
Gender distribution among respondents.

**Figure 2:**
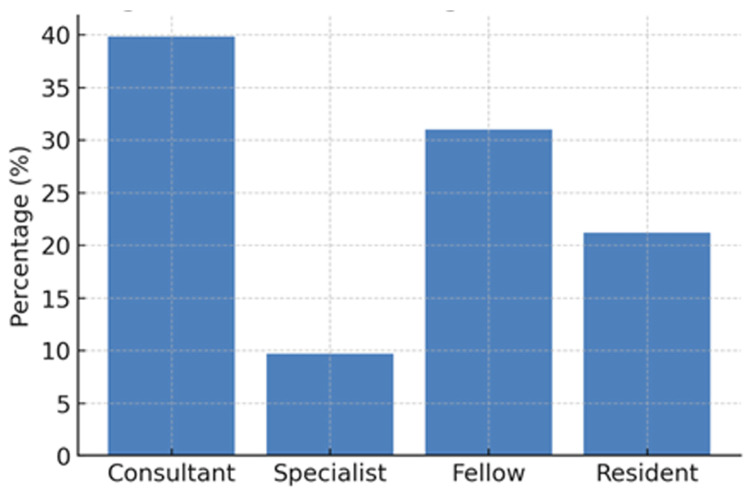
Professional grades of ophthalmologists who responded to the survey.

A total of 115 ophthalmologists participated in our study, and the response rate was 26.8%. Of these, 53 (46.1%) were male, and 62 (53.9%) were female (Figure 1). The mean age of respondents was 34.4 years (SD ± 7.6; range: 26–60), with a median of 32 years. The mean post-residency clinical experience was 4.3 years (SD ± 5.6; range: 0–30), with a median of 2 years. In terms of professional grade, 45 respondents (39.8%) were consultants, 11 (9.7%) specialists, 35 (31.0%) fellows, and 24 (21.2%) residents (Figure 2). The majority of participants had their ophthalmology practice based in Riyadh (52%), followed by Jeddah (9.6%), Alkhobar (7.8%), Dammam (7.0%), and Dhahran (7.0%).

Knowledge questions revealed variable levels of accuracy. Almost all participants correctly identified that humans do not have 50 chromosomes (92%), and 99% recognized misconceptions in statements about autosomal recessive inheritance. In addition, 86% recognized misconceptions in statements about autosomal dominant inheritance. Similarly, 85% understood that not all genetic variants cause disease. Importantly, 85% of ophthalmologists confirmed that gene therapy is no longer confined to research purposes only (85%). However, only 9% correctly answered the DNA base pairing question, and 54% recognized the maternal inheritance pattern of mitochondrial diseases. Sources of information were primarily through the internet (65%), followed by books (14%) and medical school (12%).

Confidence scores indicated moderate overall confidence. The highest mean scores were reported for referral to specialists (mean 7.3 ± 2.3) and obtaining family history (mean 6.6 ± 2.3). Clinical evaluation scored moderately (mean 5.5 ± 2.3). In contrast, confidence in deciding appropriate genetic tests (mean 4.7 ± 2.8), discussing prenatal diagnosis (mean 4.8 ± 2.8), and providing detailed counselling was low. This pattern highlights the reliance on referral networks rather than independent genetic decision-making).

**Figure 3:**
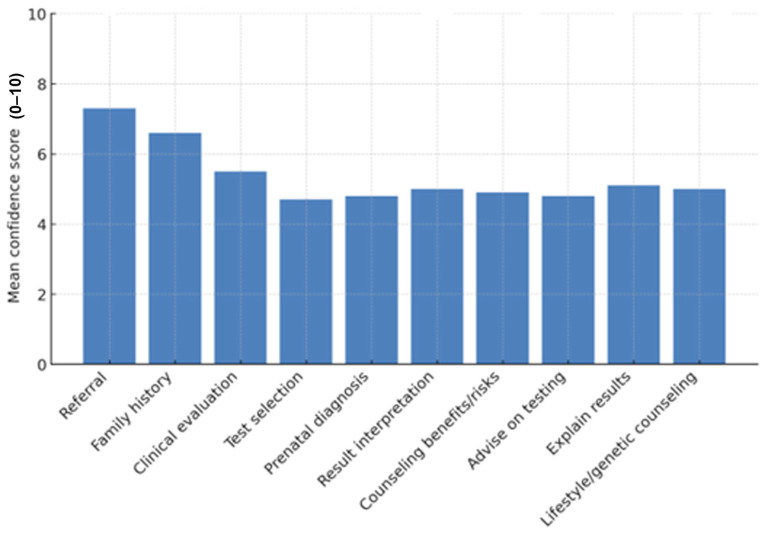
Self-rated confidence in performing clinical tasks that are related to the genomic medicine practice.

**Figure 4:**
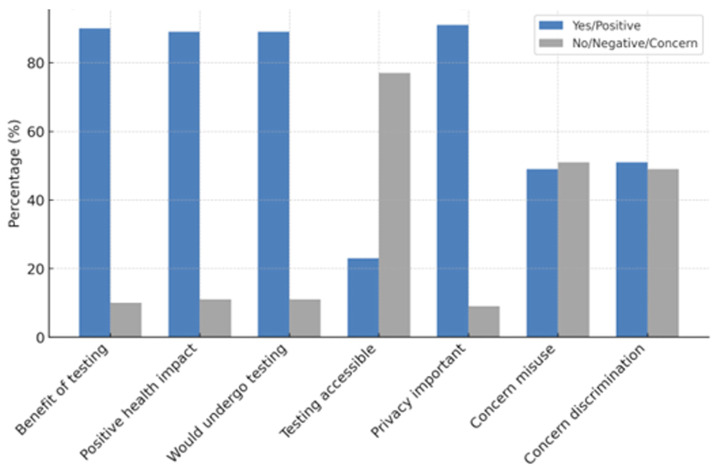
Attitudes and perceptions of ophthalmologists towards genetic testing.

The majority of participants considered genetic testing to be beneficial (90%) and reported that it has a favorable impact on health and well-being (89%). A substantial proportion (89%) were in favor of having genetic evaluations if recommended. Nonetheless, 77% believed that testing is not readily accessible to the public. Ninety-one percent displayed great agreement with the importance of confidentiality and privacy. Forty-nine percent of individuals expressed concern regarding the potential misuse of genetic data, while 51% indicated apprehension about the possibility of genetic data resulting in discrimination in employment, insurance, or healthcare. Most participants (89%) predicted that genetic testing would become more prevalent in the future. Figure 3 illustrates that individuals exhibited the highest confidence in recommending someone to an expert, while they demonstrated the lowest confidence in selecting a test or providing advice. Figure 4 illustrates that the majority of individuals perceived testing as beneficial; nonetheless, numerous concerns around privacy, accessibility, and potential misuse were prevalent.

## 4. Discussion

This study explored the knowledge, attitudes, and practices of ophthalmologists in Saudi Arabia regarding genomic medicine and genetic testing. To the best of our knowledge, this is the first study to address the gap in understanding genomic medicine and genetic testing in the field of ophthalmology in our region. Based on our survey analysis, our findings indicate significant deficiencies in knowledge, limited confidence in clinical application, and generally positive attitudes toward genetic ophthalmic illnesses. These results, when applied in the context of regional and global data across various medical specialties, suggest the need for comprehensive training, improved infrastructure, and amendments to medical school curricula to enable the effective integration of genetics into ophthalmic service and care.

Despite the advancements in the Human Genome Project [2] and the expanding global understanding and application of genomic medicine [1], our findings reveal ongoing knowledge gaps among ophthalmologists. Participants demonstrated a good understanding of inheritance patterns; yet, some errors related to molecular genetics were found, such as DNA base pairing and mitochondrial inheritance mechanisms. These results support the previous findings highlighting similar deficiencies in knowledge among medical students and pharmacists in Saudi Arabia, as well as physicians who demonstrated uncertainty in the application of pharmacogenomics despite acknowledging its clinical relevance [6,7]. These results also show that while ophthalmologists retained certain background information about human genetics, they were not exposed to more sophisticated material in molecular genetics and special types of inheritance. As only a minority (12%) retained their information from medical school, more in-depth exposure to genomic medicine during medical school, and more importantly, at later stages of their careers, is needed to expand the knowledge of ophthalmologists about genomic medicine.

The consequences of deficient knowledge about genomic medicine are particularly relevant in ophthalmology more than in other specialties. The abundance of inherited retinal diseases, which are associated with an increasing number of genetic variants in disease-causing genes [3], makes the knowledge about genomic medicine and ophthalmic genetics essential for appropriate diagnosis and referrals. The emergence of gene-based therapies further emphasizes the critical importance of early diagnosis and referrals for centers that provide genetic therapy trials.

Notably, our findings were in alignment with international studies demonstrating that non-genetic specialists in other medical specialties—including cardiologists, oncologists, and neurologists—report inadequate training and poor preparedness for genomic applications in clinical practice [13,14,15]. A comprehensive survey in Australia confirmed that while clinicians recognize genomic relevance, most feel unprepared to implement these tools without substantial additional training [15]. The low correct response rate for the DNA base pairing (9%) in our study, in contrast to the high correct responses for the numbers of chromosomes and the modes of inheritance, reflects the lack of depth in knowledge about genomic medicine despite the good understanding of the general concepts of the subject.

Confidence levels among our participants varied significantly across different genomic medicine applications. Participants expressed the highest confidence in referring patients to genetics specialists but expressed lower confidence in ordering tests independently, interpreting results, and providing genetic counseling. Similar patterns of resorting to referral pathways rather than developing one’s own genomic competencies have been widely reported in the literature [15,16,17]. Similar findings were found among cardiology and oncology studies, where clinicians admitted to avoiding direct genomic decision-making due to a lack of confidence [14]. These patterns have been observed throughout Middle Eastern and Arab countries, where clinicians might lack the appropriate exposure to genomic medicine during training and require improvements in genetic services [6,16]. Although referring patients to genetic specialists might be an appropriate decision when dealing with patients with inherited eye diseases, the referral to ophthalmic geneticists might not be easy for some patients who resort to performing genetic testing in the most accessible facility. Ophthalmologists who are confident in interpreting genetic testing might make a difference in the management of these patients through careful monitoring of patients with syndromic or nonsyndromic pathological myopia or appropriate referrals for other specialties for syndromes that involve multiple organs, like Usher syndrome and Bardet–Biedl syndrome [18]. The National Genomic Initiative might need to address these gaps in knowledge and confidence through continuous medical education activities and workshops for ophthalmologists.

Our results highlight that informal educational resources, which include online materials and presentations, served as primary sources of genomic knowledge. These findings indicate broader learning patterns among health care professionals that extend beyond structured, comprehensive education [5,6,7,16]. Such reliance on informal learning might contribute to incomplete and insufficient understanding and reinforces the critical need for specialty-specific genomic curricula.

The majority of participating ophthalmologists demonstrated positive attitudes toward genetic testing, with nearly 90% expressing willingness to undergo testing themselves if clinically advised. This optimism is supported by the recent advances and the introduction of precision medicine and individualized patient care [1,4,19]. However, participants identified substantial barriers to implementation, including limited service availability, high costs, and insufficient institutional readiness. These concerns align with issues raised throughout the Middle East [6] and persist even in Western countries where limitations in infrastructure and challenges in reimbursement remain significant obstacles [11,19].

Participants expressed significant concerns regarding data confidentiality, genetic discrimination, and the potential for misuse of genetic information. These ethical challenges were not confined to Saudi Arabia, as similar apprehensions were expressed across Canada, the United States, and Europe [10,19]. DuBois et al. specifically reported that both patients and clinicians emphasize the fundamental importance of trust, privacy safeguards, and comprehensive informed consent in the implementation of genomic medicine [19]. Knoppers and Beauvais similarly argued that ethical considerations are inseparable from successful clinical integration of genomic medicine [9].

Our findings suggest that ophthalmologists in Saudi Arabia are not yet adequately prepared for the integration of genomic medicine into their routine practice, despite recognizing its substantial clinical value. This readiness gap is particularly concerning given that precision medicine has been designated as a national health priority, with Saudi Arabia making significant investments in genomics infrastructure and workforce development [17]. For ophthalmology specifically, strengthening genomic literacy is essential to support effective integration of genomic medicine in clinical practice and optimal uptake of emerging therapies for inherited eye diseases today and in the future.

This study is the first study assessing the genetic competencies of ophthalmologists in Saudi Arabia, which provides specialty-specific evidence to the regional literature. In addition, it provides a foundation for educational and policy reforms that could enhance public preparedness for genomics. Limitations of this study include dependence on self-reported data, which may inadequately represent actual competencies, and the cross-sectional design, which restricts causal inference. About half of the study participants were from Riyadh, and this might have resulted in overestimation or underestimation of knowledge and confidence. In addition, this geographical distribution might limit the generalizability of results and might have resulted in a sampling bias. The relatively small sample size and unknown response rate further affect representativeness. Additionally, the cross-sectional design might limit the causal inference. This design cannot determine whether increased training would directly improve confidence or practice.

## 5. Conclusions

This study demonstrates that while ophthalmologists in Saudi Arabia generally recognize the importance of genomic medicine and express positive attitudes toward its use, significant knowledge gaps, limited confidence in clinical application, and systemic barriers hinder its integration into practice. These findings mirror international trends and emphasize the urgent need for structured genomic education, improved infrastructure, and clear legislative frameworks to ensure safe and effective implementation. Strengthening genomic knowledge and confidence among ophthalmologists might be critical not only for accurate diagnosis and timely referral but also for enabling the successful adoption of emerging gene-based therapies and advancing precision medicine in Saudi Arabia. Concrete steps that support this direction include higher exposure for ophthalmic genetics among ophthalmology residents, establishing multidisciplinary clinics with genetic counselors, and developing national guidelines for genetic testing in ophthalmology.

## Data Availability

All the data presented in this manuscript are available on request.

## Supplementary Materials

The following supporting information can be downloaded at: https://www.mdpi.com/article/10.3390/jpm16050267/s1, Table S1: Knowledge, Attitude and Practices towards Genomic Medicine among Ophthalmologists in Saudi Arabia.

## Abbreviations

The following abbreviations are used in this manuscript:

KAP	knowledge, attitudes, and practices

## References

[1] Collins F.S., Morgan M., Patrinos A. (2003). The Human Genome Project: Lessons from large-scale biology. Science.

[2] Daiger S.P., Sullivan L.S., Bowne S.J. (2013). Genes and mutations causing retinitis pigmentosa. Clin. Genet..

[3] Haga S.B., O’Daniel J.M., Tindall G.M., Lipkus I.R., Agans R. (2012). Survey of US public attitudes toward pharmacogenetic testing. Pharmacogenom. J..

[4] Olwi D., Merdad L., Ramadan E. (2016). Knowledge of Genetics and Attitudes toward Genetic Testing among College Students in Saudi Arabia. Public Health Genom..

[5] Alzoubi A., Kanaan H., Alhazaimeh D., Gharaibeh S., Mukattash T.L., Kheirallah K. (2020). Knowledge, attitude, future expectations, and perceived barriers of medical students and physicians regarding pharmacogenomics in Jordan. Int. J. Clin. Pract..

[6] Alrabiah Z., Syed W., Babelghaith S.D., Al Arifi M.N. (2023). Clinical Knowledge, Attitude, and Perceptions of Community Pharmacists Towards Pharmacogenomics—A Cross-Sectional Study from Saudi Arabia. Pharmacogenom. Pers. Med..

[7] Alsanosi S.M., Al Eissa M.M. (2025). Awareness of pharmacogenomic capabilities among physicians and pharmacists in Saudi Arabia within current clinical practice. J. Umm Al-Qura Univ. Med. Sci..

[8] AlEissa M.M., Alhawsawi A.A., Alonazi R., Magharbil E., Aljahdali A., AlBalawi H.B., Alali N.M., Hameed S., Abu-Amero K.K., Magliyah M.S. (2025). Advances in Precision Therapeutics and Gene Therapy Applications for Retinal Diseases: Impact and Future Directions. Genes.

[9] Knoppers B.M., Beauvais M.J.S. (2021). Three decades of genetic privacy: A metaphoric journey. Hum. Mol. Genet..

[10] Manolio T.A., Chisholm R.L., Ozenberger B., Roden D.M., Williams M.S., Wilson R., Bick D., Bottinger E.P., Brilliant M.H., Eng C. (2013). Implementing genomic medicine in the clinic: The future is here. Genet. Med..

[11] AlRasheed M.M., AlAli H., Alsuwaid A.F., Khalaf S., Ata S.I., BinDhim N.F., Bakheet D., Khurshid F., Alhawassi T.M. (2021). Gene Therapy Knowledge and Attitude Among Healthcare Professionals: A Cross-Sectional Study. Front. Public Health.

[12] Yu M.W.C., Fung J.L.F., Ng A.P.P., Li Z., Lan W., Chung C.C.Y., Li Y., Liu Y., Chung B.H.Y., Wong W.C.W. (2021). Preparing for the genomic revolution: Attitudes, clinical practice, and training needs in delivering genetic counseling in primary care in Hong Kong and Shenzhen, China. Mol. Genet. Genom. Med..

[13] Chow-White P.A., Ha D., Laskin J. (2017). Knowledge, attitudes, and values among physicians working with clinical genomics: A survey of medical oncologists. Hum. Resour. Health.

[14] Christensen K.D., Vassy J.L., Jamal L., Lehmann L.S., Slashinski M.J., Perry D.L., Robinson J., Blumenthal-Barby J., Feuerman L., Murray M. (2016). Are physicians prepared for whole genome sequencing? A qualitative analysis. Clin. Genet..

[15] Haga S.B., Kim E., Myers R.A., Ginsburg G.S. (2019). Primary care physicians’ knowledge, attitudes, and experience with personal genetic testing. J. Pers. Med..

[16] Mawkili W.A. (2025). The future of personalized medicine in Saudi Arabia: Opportunities and challenges. Saudi Med. J..

[17] AlEissa M.M., Alhawsawi A.A., Milibari D., Schatz P., AlBalawi H.B., Alali N.M., Abu-Amero K.K., Hameed S., Magliyah M.S. (2025). Genetics and Clinical Findings Associated with Early-Onset Myopia and Retinal Detachment in Saudi Arabia. Genes.

[18] DuBois J.M., Callier S.L., Garrison N.A., James C.A., Henderson G.E. (2021). Attitudes and expectations toward genomics and precision medicine: Perspectives from medical professionals and patients. BMC Med. Ethics.

[19] Katsanis S.H., Katsanis N. (2013). Molecular genetic testing and the future of clinical genomics. Nat. Rev. Genet..

