# Supplementary material for: Genomic Medicine Among Ophthalmologists: Knowledge, Current Practice, and Barriers
Source: J Pers Med. 2026 May 16;16(5):267. doi: 10.3390/jpm16050267 (PMC13208326; doi:10.3390/jpm16050267)
Supplement: Supplementary file 1 [file jpm-16-00267-s001.zip › jpm-4248586-supplementary.pdf]

Data Collection Sheet – Research Department - KKESH

Project No.: ____________________

Project Title: Knowledge, Attitude and Practices towards Genomic Medicine among Ophthalmologists in Saudi Arabia

You are invited to participate in the above-stated study, which is aimed at [XXXXXXXXXX].

By answering this questionnaire, you voluntarily provide your consent to participate in the study and permit the researcher to utilize the information obtained, fully understanding that your privacy and confidentiality are well preserved.

Should you have any questions regarding the study, please feel free to contact [name/contact].

**Demographics**				
1- Gender	Male		Female	
2- Age				
3- Years of Post-board Experience	< 5 y	5 – 10 y	>10 y	
4- Ophthalmology grade (Resident / Fellow / Specialist / Consultant)	Resident	Fellow	Specialist	Consultant
5- City of main practice				
6- Undergraduate genomic medicine education	Extensive	Intermediate	Low	
7- Postgraduate genomic medicine training	Extensive	Intermediate	Low	
8- Working Organization	Government	Private	Charity	

**Knowledge**				
1- Humans have 50 chromosomes.	Yes	No	May be	
2- What is the main source of information you used to learn about genetic diseases and ophthalmic genetics	Internet	Colleagues	Friends	Other
3- What is your level of awareness on how genetic diseases are inherited and passed down in families?	Fully aware	Intermediate awareness	Low awareness	Not aware
4- Adenine (A) only pairs with cytosine (C) and Thymine (T) only pairs with Guanine (G).	Yes	No	I do not know	
5- All identified genetic variants cause diseases	True	False	I do not know	
6- Gene therapy is currently available in research setting only	True	False	I do not know	
7- In Autosomal recessive inheritance, all offspring will be affected	True	False	I am not sure	
8- In Autosomal dominant inheritance, one of the parents has to be affected	True	False	I am not sure	

9- In X-linked dominant inheritance, girls will always be affected	True	False	I am not sure
10-In mitochondrial diseases, males and females have equal chances to inherit the disease-mutation	True	False	I am not sure

**Self-rated confidence score**	
1- Obtain information about genetic disorders from family history	1 - 2 - 3 - 4 - 5 - 6 - 7 - 8 - 9 - 10
2- Clinical evaluation of genetic disorders	1 - 2 - 3 - 4 - 5 - 6 - 7 - 8 - 9 - 10
3- Referral to a relevant specialist for suspected genetic disorders	1 - 2 - 3 - 4 - 5 - 6 - 7 - 8 - 9 - 10
4- Decide which genetic testing should be done for suspected genetic disorders	1 - 2 - 3 - 4 - 5 - 6 - 7 - 8 - 9 - 10
5- Discuss issues related to prenatal diagnosis with your patients	1 - 2 - 3 - 4 - 5 - 6 - 7 - 8 - 9 - 10
6- Assess if the patient's genetic test results are meaningful	1 - 2 - 3 - 4 - 5 - 6 - 7 - 8 - 9 - 10
7- Discuss the benefits, risks, and limitations of genetic testing with patients	1 - 2 - 3 - 4 - 5 - 6 - 7 - 8 - 9 - 10
8- Discuss and counsel patients on whether they should get a genetic test	1 - 2 - 3 - 4 - 5 - 6 - 7 - 8 - 9 - 10
9- Explain to patients on genetic testing results and provide genetic counselling	1 - 2 - 3 - 4 - 5 - 6 - 7 - 8 - 9 - 10
10- Provide counselling on genetic screening strategies and lifestyle changes	1 - 2 - 3 - 4 - 5 - 6 - 7 - 8 - 9 - 10

**Genetic Testing for existing eye disease(s)**				
Are you familiar with the concept of genetic testing?	Familiar	Moderately familiar	Low familiarity	Not familiar
Do you see a benefit in someone undergoing genetic testing?	Yes	No	Not sure	
Are there any concerns or drawbacks that you associate with genetic testing?	Yes	No	Not sure	
	If yes, what concern/drawback: ……………………………………………			
Do you think genetic testing will have a positive impact on a person's health and well-being?	Yes	No	Do not know	
Would you personally consider getting a genetic test, if advised?	Yes	No	Not Sure	
Do you think genetic testing services are easily available to the general public?	Yes	No	I do not know	
Privacy and confidentiality of genetic information is important	Strongly agree	Moderately Agree	Disagree	
Do you have any concerns about the potential misuse of genetic testing data?	Yes	No	Do not know	
Do you think genetic testing will become more prevalent in the future?	Yes	No	Do not know	
Do you have concerns or worries about the potential use of genetic information in areas like employment, insurance, or healthcare?	Yes	No	Not sure	

Filled By: ______________________ Sequence # ______________________
